# Partially Deacetylated and Fibrillated Shrimp Waste-Derived Chitin as Biopolymer Emulsifier for Green Cutting Fluids—Towards a Cleaner Production

**DOI:** 10.3390/polym14030525

**Published:** 2022-01-28

**Authors:** Oscar Aguilar-Rosas, Stephany Blanco, Mariana Flores, Keiko Shirai, Leonardo Israel Farfan-Cabrera

**Affiliations:** 1 Tecnologico de Monterrey, Escuela de Ingeniería y Ciencias, Ave. Eugenio Garza Sada 2501, Monterrey 64849, NL, México; a01731167@tec.mx; 2 Biotechnology Department, Laboratory of Biopolymers and Pilot Plant of Bioprocessing of Agro-Industrial and Food By-Products, Universidad Autónoma Metropolitana, Av. San Rafael Atlixco No. 186, Iztapalapa, Mexico City 09340, Mexico; yuru34hu@hotmail.com (S.B.); mary.flores32662@gmail.com (M.F.)

**Keywords:** metalworking fluid, cutting process, Pickering emulsion, chitin, green fluid, green manufacturing, biodegradable oil, minimum quantity lubrication

## Abstract

Up to date, most metalworking fluids (MWFs) are emulsions made of petroleum-derived oil bases and sodium petroleum sulphonate emulsifiers. They are not readily biodegradable, and their waste is hazardous for users and the environment. Therefore, green MWFs are required for achieving cleaner production processes. Recently, various MWFs have been developed using vegetable oil bases to meet biodegradability to some extent. However, the emulsifier has been scarcely replaced by a green product. This research aims to produce and evaluate Pickering emulsions made of Jatropha oil (JO) and partially deacetylated and fibrillated chitin (PDFC) as emulsifiers at different concentrations. JO is a non-edible biodegradable oil with remarkable lubricity properties, while PDFC is produced by extracting chitin from waste heads and shells of the shrimp species *Litopenaeus vannameii*, followed by partial deacetylation and further fibrillation, which improves wettability and stabilization. The prepared emulsions were characterized in terms of creaming index and size of emulsion droplets and evaluated as MWFs in actual turning operations of AISI 1018 steel bars via minimum quantity lubrication (MQL) technique. The findings suggest PDFC as a potential eco-friendly emulsifier to form green MWFs with acceptable stability generating low cutting forces and significant workpiece finishing and chips quality.

## 1. Introduction

Recently, there has been huge interest in the utilization of natural waste products and biopolymers, namely shrimp, seeds, soybean meal, etc. [1,2,3,4], to replace conventional products that cause pollution and damage to the environment by their production, use and waste. In this sense, chitin is an insoluble copolymer of *N*-acetylglucosamine, and glucosamine is an abundant natural biodegradable polymer on earth. It is present in the structural components of arthropod exoskeletons or in the cell walls of fungi and yeast [5]. Typically, it is extracted from crustacean wastes (e.g., shrimp or crab) either by thermochemical or biological treatments [6]. The latter has emerged as a green, cleaner, eco-friendly and economical process since it reduces or avoids the use of highly concentrated reagents and produces chitins with high molecular weight and crystallinity. To improve chitin solubility, it is deacetylated by the alkali method to produce chitosan-augmented free amino groups [7]. Apart from being a food-grade material, biodegradable, biocompatible and nontoxic, some chitin-derived nanomaterials have been proven as good stabilizers for high internal phase Pickering emulsions [8,9,10,11,12]. For example, individual nanofibril-like chitin is usually assembled in bundles via strong hydrogen bonding. It can be isolated through partial deacetylation and subsequently mechanical nanofibrillation to obtain positively charged chitin nanofibrils, which have an increased wettability at the interface, favoring Pickering stabilization [10]. Pickering stabilization by biobased particles and colloids is effective in preventing droplet coalescence due to a strong interfacial mechanical barrier generated by the particles, which promotes even better stability at a relatively low concentration of particles than surfactants [13]. Thus, chitin-based Pickering emulsions can be used in a wide range of industrial applications requiring good stabilization, accessibility, cost-efficiency, abundance and green practices.

The metalworking industry is one of the most demanding sectors, requiring huge quantities of emulsions technically called metalworking fluids (MWFs). MWFs, also named cutting fluids, are liquid substances used to lubricate and cool down the workpiece/cutting tool interface during machining processes. Commercial MWFs are produced either using straight oils, water-miscible oils/emulsions (WMOs) and semi-synthetic or synthetic oils. However, WMOs are the most used in about 70% of the machining processes. Emulsifiers in certain concentrations (10–15 wt%) are used commercially to disperse the oil in water by binding them together, and thus, forming stable water-miscible oils (WMOs) emulsions in a 1:5 to 1:50 oil-to-water ratio. Thus, emulsifiers are critical for achieving emulsion stability but also contribute to the reduction in cutting forces, cutting temperatures, tool wear and workpiece surface roughness depending on the emulsifier type and concentration [14,15]. The most used emulsifier for this purpose is sodium petroleum sulphonate [14]. However, other alternative emulsifiers, including nonionic emulsifiers based on linear alkylbenzene sulfonic acid, maleic anhydride esters and adduct with oleic acid and ester and ethylene oxide derived from local raw materials, are other options [16].

Currently, the renewal of metalworking processes and the establishment of environmentally friendly operations ask for eco-friendly emulsions to approach the green manufacturing demands [17]. It is well known that WMOs made of mineral oils and sodium petroleum sulphonate are not readily biodegradable, and their waste is hazardous [18]. Moreover, they are very susceptible to bacterial contamination due to large hydrocarbon amounts present in mineral oil and emulsifiers. Continuous exposure to these fluids serves as nourishment for bacteria [19], and consequently human occupational health disorders (cancers, dermatitis, lung disorders, etc.) [20]. Considering the health risks and the current and growing demands for environmental care, WMO formulations must be reworked. This has promoted the development of a great variety of eco-friendly (biodegradable and non-hazardous waste) MWFs/WMOs based on different vegetable oils (i.e., soybean oil, jatropha oil (JO), coconut oil, castor oil, palm oil, etc.) to contribute to the needs of modern green manufacturing, as reported in a vast amount of literature [21,22,23,24,25]. Nevertheless, almost all the reported documents are focused on the replacement of mineral oil with certain vegetable oils, while the emulsifier, which is frequently the root-cause of several health and environmental issues, has rarely been replaced. A recent attempt to change the conventional emulsifier of vegetable-oil-based WMOs with a green emulsifier has been presented by Srikant and Ramana [26]. They used an emulsifier derived from coconut oil called CocAmidoPropylBetaine (CAPB) to prepare sesame-oil-based WMOs. Various concentrations (5, 10, 15, 20 and 25 wt%) of the CAPB emulsifier were employed to formulate different WMOs. The performance of the formulated WMOs was tested according to cutting forces, cutting temperatures, tool wear and surface roughness of the machined surface in turning operations. The results demonstrated that the formulated WMO with 10% CAPB emulsifier content exhibited performance (in terms of cutting temperatures, tool wear and surface roughness) similar to a regular commercial WMO. Thus, they pose CAPB as a potential eco-friendly alternative to conventional emulsifiers.

Considering the potential of chitin-derived nanomaterials as an emulsifier for producing green emulsions with good stability, as well as the scarcity of proven green emulsifier options to produce WMOs, this work looks at replacing both the mineral oil and conventional emulsifier with more eco-friendly and sustainable alternatives, such as non-edible JO as the base oil and partially deacetylated and fibrillated chitin (PDFC) as an emulsifier. The novelty of the present work is the utilization of PDFC as an eco-friendly emulsifier to produce green WMOs as MWFs for the metalworking industry. This exploratory research is a pioneer in the application of PDFC as an emulsifier for MWFs. The Pickering chitinous emulsifier used in this work was produced from shrimp waste by biological extraction and deacetylation. The new class of WMOs (emulsions) were prepared at several concentrations of JO and PDFC and tested in terms of their performance in machining by measuring cutting forces, cutting temperature, the surface roughness of the machined surface and chip formation in turning operations under the minimum quantity lubrication technique (MQL).

## 2. Materials and Methods

### 2.1. Chitin Production and Emulsions Preparation

Heads and shells of the shrimp species *Litopenaeus vannameii* were kindly supplied by Netmar (Mexico City, Mexico). The food waste was generated after the separation of the edible parts, and it was shipped to the laboratory (−9 °C). Later on, it was crushed in a meat grinder (Torrey 32-3, Mexico City, Mexico) and stored at −20 °C until use.

The shrimp waste was thawed and mixed with sucrose (10 wt/wt%) and 24 h culture of *Lactobacillus brevis* in Man Rogosa Sharpe broth at 30 °C as a starter (5 vol/wt%). In total, 8 kg of this mixture was placed into a 10 kg column reactor [27]. The reactor was incubated up to 120 h at 30 °C. The fermented solid was treated with HCl (0.25 N at 25 °C) and NaOH (0.25 N at 25 °C) for 1 h in each step and decolored with ethanol (96 vol/wt%). Chitins were deacetylated with NaOH (30 wt%). Followed by aqueous neutralization, samples were dried for further characterization. Partially deacetylated chitin (1 wt%) was dispersed in an acetic acid solution (0.1 M) and fibrillated by high-speed blending. The suspension of partially deacetylated and fibrillated chitin (PDFC) was characterized through chemical analysis; molecular weight by intrinsic viscosity determination; and the degree of acetylation (DA) by proton nuclear magnetic resonance (^1^HNMR) spectroscopy in a Bruker AVANCE-III 500 (Agilent Technologies, Inc., Santa Clara, CA, USA) spectrometer at 200 MHz in DCl/D_2_O with 3-(trimethylsilyl) propionic acid as the internal reference. The transmittance of deacetylated chitin and PDFC suspensions were measured in a range of 200 and 800 nm in a UV–Vis spectrophotometer (Thermo Scientific, Waltham, MA, USA).

The creaming index (*CI*) determined the gravitational stability, which was carried out by centrifugation (4000× *g* for 15 min) to accelerate the rate of creaming [28]. *CI* was determined considering the height of the serum layer (*H_S_*) and the total height (*H_t_*) by Equation (1). *CI* determinations were carried out in triplicate.

(1) CI(%)=HSHt×100

The emulsion was diluted 1:10 in an acetic acid solution (0.1 M) and placed on a glass slide and observed in an optical microscope (Carl Zeiss AG, Oberkochen, Germany). The microscope was coupled to a digital camera that captures the images. Three-hundred different digital images were taken and analyzed by Image J 1.51k to obtain the diameter of the emulsion droplets and their frequencies.

JO was the vegetable oil base selected to produce the emulsions. The characterization of the fatty acids profile of JO has been reported elsewhere [29,30]. It contains about 32.7% of arachidonic acid, 26.6% of linoleic acid, 25.2% of behenic acid, 11.2% of oleic acid and 4.2% of lauric acid. This non-edible oil is currently considered one of the most promising alternatives as a biolubricant for a wide range of applications due to several agronomical advantages [31] and suitable tribological properties, as demonstrated in previous works [29,30]. The dispersed phase of JO was dripped to the continuous phase (PDFC solutions) and homogenized at 9500 rpm for 7 min and 13,500 rpm for 3 min with an Ultra Turrax T-25 homogenizer (Janke and Kunkel GmbH and Co., Staufen, Germany). The concentration of PDFC was used in four levels of 0.25, 0.5, 0.75 and 1 wt% and for JO at three levels of 10, 15 and 20 wt%.

### 2.2. Machining Testing

In this study, the cutting performance of twelve emulsions made of various concentrations of JO (10, 15 and 20 wt%) and the PDFC emulsifier (0.25, 0.5, 0.75 and 1 wt%), which will be named in the following as JO + PDFC emulsions, were tested. Moreover, they were compared, as a reference, to a commercial semisynthetic oil-based MWF emulsion (CIMSTAR^®^ 60 at 10 wt%) containing sodium alkyl aryl sulfonate emulsifier and to dry machining in turning operations.

**Figure 1:**
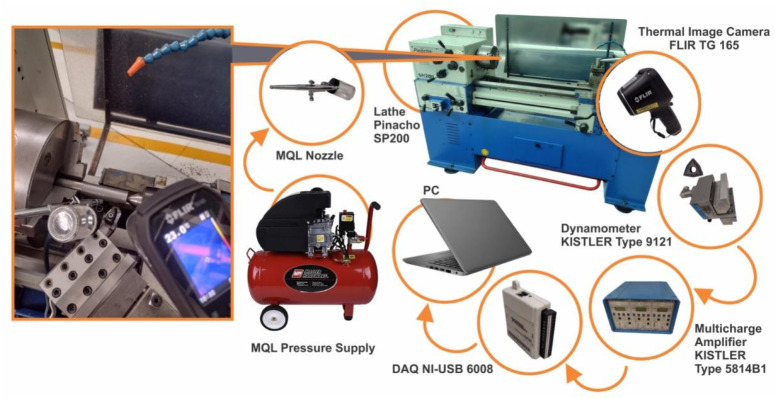
Machining (turning operation) set-up.

AISI 1018 steel bars (C-0.15e0.20%, Mn-0.60e0.900%, S-0.050%, P-0.050%, hardness-116 ± 2 HB) of size Ø25.4 × 120 mm were machined on a 5.5 HP lathe (Pinacho SP 200) under constant cutting parameters of 70 m/min as the cutting speed, 860 RPM, 0.15 mm/rev as feed and 0.5 mm as the cutting depth. The schematic diagram of the cutting (turning) test set-up is illustrated in Figure 1. The range of the cutting conditions was selected based on the recommendations of the tool supplier for finishing operations. A brand-new coated carbide insert was used to test each emulsion, and WNmG 080404e-Fm Grade T9325 (Pramet, Šumperk, Czech Republic) (Tool Signature—inclination angle: −6°, orthogonal rake angle: −6°, orthogonal clearance angle: 6°, auxiliary cutting edge angle: 10°, principal cutting edge angle: 80°, nose radius: 0.8 mm) was used as the cutting tool. The corresponding tool holder DWLNR 2020K 08 was used. Each emulsion was agitated before the machining testing. The emulsion flow was directed to the flank face with a constant flow rate of 4 × 10^−5^ m^3^/h via the MQL technique with constant parameters of air pressure, nozzle distance and nozzle diameter of 0.4 MPa, 20 mm and 0.4 mm, respectively. The MQL technique is one of the most promising green machining techniques that can yield a reduction in the consumption of MWF (by more than 90%) while ensuring the surface quality and tool life by applying only the amount of MWF needed to lubricate the cutting interface. The cutting forces (cutting force (Fx), radial force (Fy) and feed force (Fz)) were measured using a piezo-electric dynamometer (Kistler Type 9121), a dual model amplifier (Kistler type 5814B1) and a data acquisition device (NI-USB 6008) along with a custom Lab-View program. The cutting temperatures were measured using a thermal image camera (FLIR TG 165) during the whole test. The workpiece surface finish was measured in terms of surface roughness, Ra, using a roughness meter (Surfcom 130A) after each cutting operation. Roughness was measured in the longitudinal direction along the machined area of each workpiece taking the average of 3 measurements with a 120° offset from the previous measure. Each emulsion was tested three times at the same conditions, and the data were statistically analyzed.

### 2.3. Statistical Analysis

The NCSS program version 7.0 (NCSS LLC., Kaysville, UT, USA) was used to determine the significance among the emulsion formulation on CI, cutting forces, cutting temperature and surface roughness studied. The means of the results were compared with Tukey–Kramer multiple means comparison test (*p* ≤ 0.05).

## 3. Results and Discussion

### 3.1. Characterization of the Formulated Emulsions

**Figure 2:**
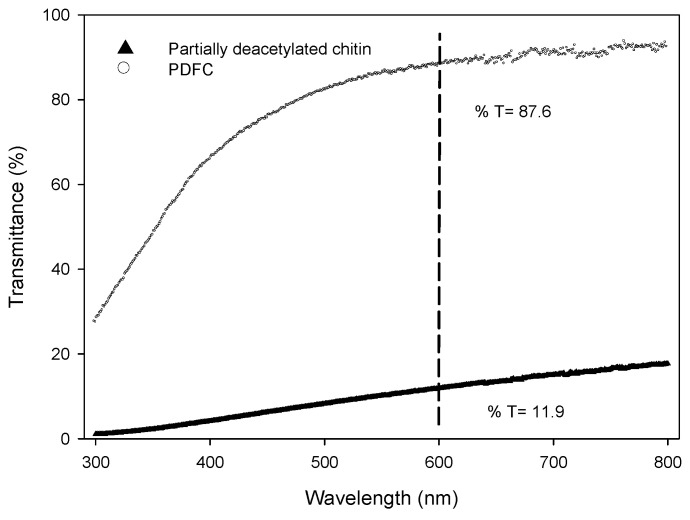
Transmittance of partially deacetylated chitin and PDFC suspensions.

The partially deacetylated chitin was extracted from a lactic acid fermentation, followed by chemical purification, achieving chitin almost free of minerals and proteins with deproteinization and demineralization degrees of 99.98% and 99.97%, respectively. The viscosimetric molecular weight was 150.62 kDa, and the degree of acetylation was 25%. A degree of acetylation below 50% disrupts the crystalline structure by electrostatic repulsions and promotes its solubilization in water. The final pH of the emulsions was in the range of 3.5–4.5, which allows amino groups to be positively charged, enhancing the hydrophilicity and the interaction with the aqueous phase; in contrast, the *N*-acetyl groups for their partial hydrophobicity might be adsorbed at oil–water interfaces that promote emulsion stabilization. The mechanical disintegration in the partially deacetylated chitin acidic solution was enough to break the hydrogen bonds between the nanofibrils; the transmittance is presented in Figure 2 as evidence of this dispersion. PDFC showed a higher transmittance of 87.6% at 600 nm than the partially deacetylated chitin (11.9%) as a result of a reduction in the mean diameter of fiber for the fibrillation process.

**Figure 3:**
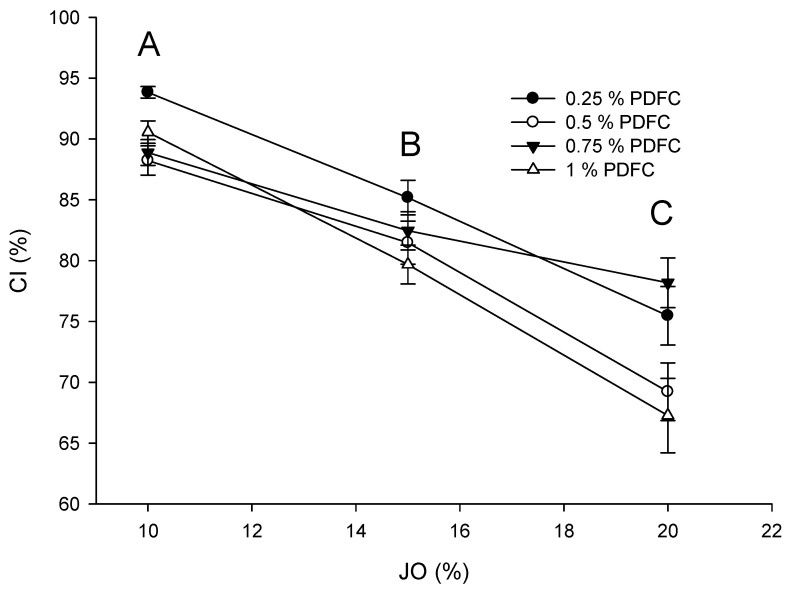
Accelerated assay for determination of creaming index of the emulsions of JO and PDFC. JO concentrations with different letters (A, B or C) were significantly different from each other (*p* ≤ 0.05) according to Tukey–Kramer’s multiple means comparison test.

**Figure 4:**
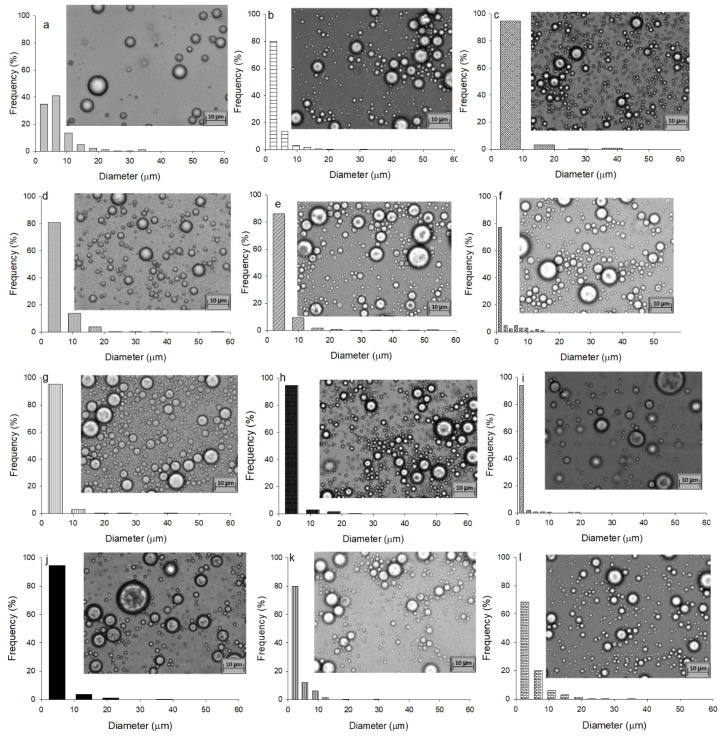
Droplet of emulsions size distribution of the emulsions formulated with JO and PDFC determined by microscopic observation (100×): 10% JO-0.25% PDFC (**a**); 10% JO-0.5% PDFC (**b**); 10% JO-0.75% PDFC (**c**); 10% JO-1% PDFC (**d**); 15% JO-0.25% PDFC (**e**); 15% JO-0.5% PDFC (**f**); 15% JO-0.75% PDFC (**g**); 15% JO-1% PDFC (**h**); 20% JO-0.25% PDFC (**i**); 20% JO-0.5% PDFC (**j**); 20% JO-0.75% PDFC (**k**); and 20% JO-1% PDFC (**l**).

Figure 3 shows the CI determined for the emulsions. Only the amount of JO was significantly different (*p* ≤ 0.05): as the JO concentration rises, the CI decreases. The size distribution of the emulsions was monomodal, and the highest frequency was found at smaller sizes (<10 mm), as shown in Figure 4. The increase in JO concentration reduced the frequency of large sizes in the emulsion. The plausible explanation is the interaction of the amine group with the carboxyl groups of the fatty acids of JO, as, in water, the pKa is 4.75 [32], therefore promoting ionic gelation. Since the pH of JO is 9, as JO increases in the emulsion, so does the pH (4.5), but there is also interplay among intermolecular strong electrostatic repulsion between the positively charged chitosan in the acidic solution, thus promoting PDFC solubility. In addition, the *N*-acetyl groups contribute to the emulsion stability for the steric hindrance that could avoid the flocculation [33]. However, when the concentration of PDFC and JO increased 1% and 20%, respectively, the frequency of larger-sized droplets augmented than that at lower concentrations. This might be due to the interaction between free PDFC with the droplets, which promoted the clumping of droplets by flocculation. Moreover, the augmented frequency of large size droplets might be caused to the JO dispersed and PDFC depletion. Despite the instability determined with the accelerated stability test, the emulsions were easily suspended before use on the application in the turning operations and remained in a single layer for at least 12 h.

### 3.2. Machining Performance

#### 3.2.1. Cutting Forces

**Figure 5:**
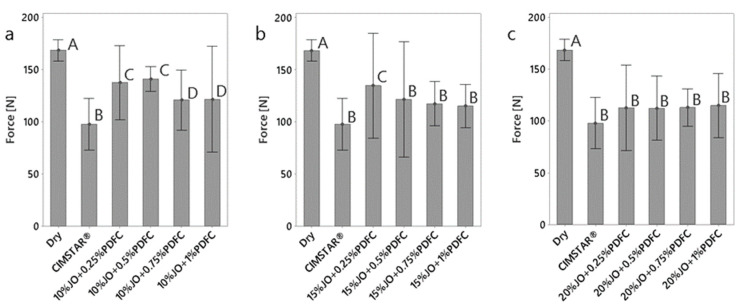
Comparison of cutting force (Fz) obtained for dry conditions and the emulsions tested: (**a**) emulsions with 10 wt% of JO; (**b**) emulsions with 15 wt% of JO; (**c**) emulsions with 20 wt% of JO. The histograms with the same letter (A, B, C or D) were not significantly different from each other (*p* ≤ 0.05) according to Tukey–Kramer’s multiple means comparison test. Error bars represent the 95% confidence interval (corresponding to a ±2.5 of the standard deviation) of the mean.

**Figure 6:**
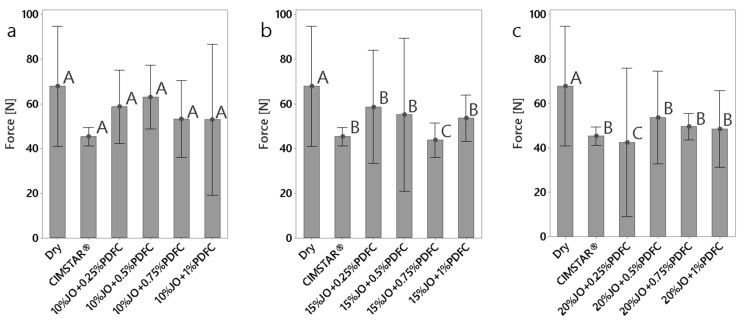
Comparison of radial force (Fy) obtained for dry conditions and the emulsions tested: (**a**) emulsions with 10 wt% of JO; (**b**) emulsions with 15 wt% of JO; (**c**) emulsions with 20 wt% of JO. The histograms with the same letter (A, B or C) were not significantly different from each other (*p* ≤ 0.05) according to Tukey–Kramer’s multiple means comparison test. Error bars represent the 95% confidence interval (corresponding to a ±2.5 of the standard deviation) of the mean.

**Figure 7:**
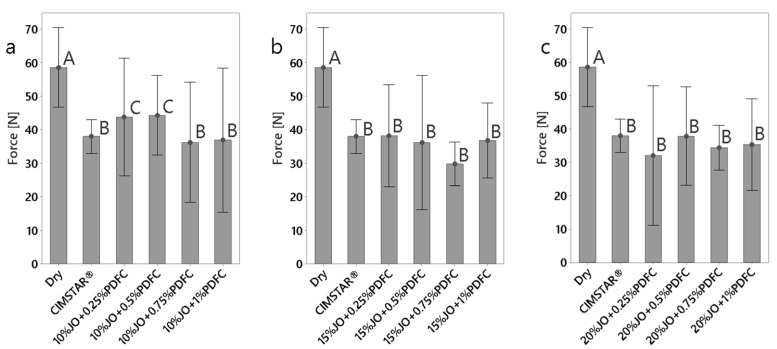
Comparison of feed force (Fx) obtained for dry conditions and the emulsions tested: (**a**) emulsions with 10 wt% of JO; (**b**) emulsions with 15 wt% of JO; (**c**) emulsions with 20 wt% of JO. The histograms with the same letter (A, B, or C) were not significantly different from each other (*p* ≤ 0.05) according to Tukey–Kramer’s multiple means comparison test. Error bars represent the 95% confidence interval (corresponding to a ±2.5 of the standard deviation) of the mean.

The obtained forces (cutting, feed and radial) are presented in Figure 5, Figure 6 and Figure 7, respectively. According to the three cutting force components, the cutting force (Fz) and radial force (Fx) are the largest and smallest in magnitude, respectively. It was found that all the JO + PDFC emulsions and the commercial MWF (CIMSTAR^®^) exhibited lower forces (in the three force components) than dry machining. This was expected since emulsions act as a lubricant, generating a protective layer around the tooltip and the workpiece to reduce friction, to some extent, during the cutting process [34]. Comparing the cutting forces of the JO + PDFC emulsions to those of the commercial semisynthetic MWF (CIMSTAR^®^), the latter exhibited the lowest cutting force. This is associated with the complex formulation of commercial MWF’s that involves additives (e.g., extreme pressure additives, friction modifiers, etc.), which help to reduce friction more efficiently. In the case of the JO + PDFC emulsions, the cutting forces tended to reduce with the increase in JO concentration. It is ascribed to the formation of more consistent lubricating layers on the cutting tool. Additionally, the cutting forces varied significantly with the PDFC content, especially for the two lowest JO concentrations (10 and 15 wt%). In the case of the largest JO concentration (20 wt%), the cutting forces were almost similar for all the PDFC concentrations. This can be associated with the better emulsion stability and larger droplet size produced by any PDFC content added in these emulsions. The variation in PDFC content did not have a significant effect on the cutting forces. It is noteworthy that all the emulsions were sufficiently agitated before being used in the machining test as a typical practice in conventional cutting processes. Thus, it is argued that the separation of the emulsion did not play a significant role in the cutting results. Thus, according to the cutting force results for the JO + PDFC emulsions, the best performance as cutting fluid was obtained by using 20 wt% of JO with any concentration of PDFC emulsifier.

#### 3.2.2. Cutting Temperatures

**Figure 8:**
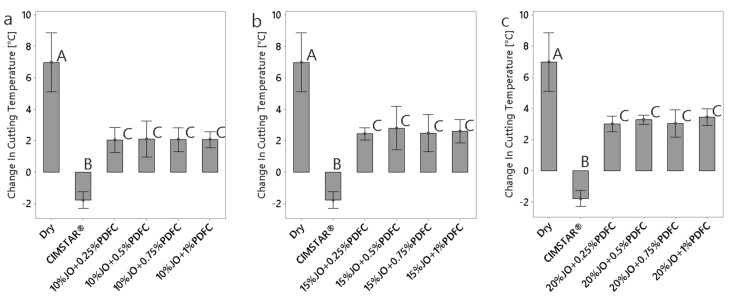
Comparison of cutting temperature change obtained for dry condition, CIMSTAR^®^ and the JO + PDFC emulsions: (**a**) emulsions with 10 wt% of JO; (**b**) emulsions with 15 wt% of JO; (**c**) emulsions with 20 wt% of JO. The histograms with the same letter (A, B, or C) were not significantly different from each other (*p* ≤ 0.05) according to Tukey–Kramer’s multiple means comparison test. Error bars represent the 95% confidence interval (corresponding to a ±2.5 of the standard deviation) of the mean.

Heat is generated during machining by two mechanisms: the first one is caused by the shear and plastic deformations suffered by the workpiece when the material is removed, while the second is caused by friction between the tool-tip and the workpiece. Figure 8 shows a comparison of the total temperature change at the tool-tip interface during the turning operations at different machining conditions (dry machining, using CIMSTAR^®^ and all the JO + PDFC emulsions). The dry machining condition presented the highest heating, producing a change in temperature of about +7 °C; meanwhile, the use of CIMSTAR^®^ generated the best cool down (a temperature change of about −2 °C). Although the use of all the JO + PDFC emulsions did not reduce the temperature as effectively as the commercial MWF, they promoted minimal temperature change (from +2 up to +3.5 °C), which is positive for turning processes. Moreover, it was found that the temperature change increases with the increase in JO contained in the emulsions; the larger content of oil, the shorter content of water (coolant) in the emulsion. The concentration of PDFC had no significant effect on the temperature change for any of the JO + PDFC emulsions.

#### 3.2.3. Surface Finish

**Figure 9:**
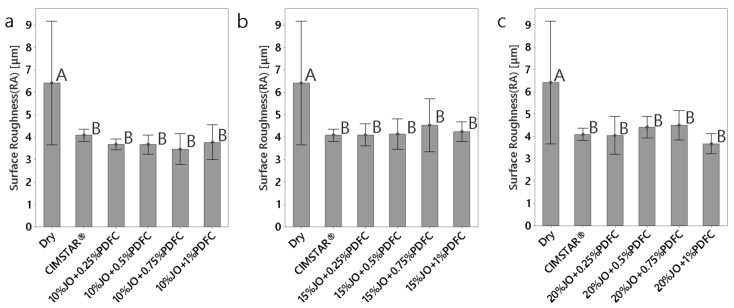
Comparison of surface finishing in terms of roughness (Ra) obtained for the machined workpieces (AISI 1018) under dry condition and using the different emulsions: (**a**) emulsions with 10 wt% of JO; (**b**) emulsions with 15 wt% of JO; (**c**) emulsions with 20 wt% of JO. The histograms with the same letter (A or B) were not significantly different from each other (*p* ≤ 0.05) according to Tukey–Kramer’s multiple means comparison test. Error bars represent the 95% confidence interval (corresponding to a ±2.5 of the standard deviation) of the mean.

In any machining process, the less rough, the better the finish of the workpiece. It is always a result desired since lower values of surface roughness on machined components offer low subsurface and residual stresses, which result in a greater overall structural integrity and aesthetic characteristic of the component [35]. Figure 9 shows the roughness, Ra, obtained after the turning operations for the machined steel workpieces under dry conditions using CIMSTAR^®^ and using all the JO + PDFC emulsions. As was expected, the dry cutting conditions generated the highest value of surface roughness (about 6.42 μm). This condition produced the highest cutting forces, which promote high levels of vibrations on the tool resulting in an uneven cutting profile and, consequently, high surface roughness. In contrast, using the CIMSTAR^®^ and all the JO + PDFC emulsions, the roughness was reduced to about 3.5–4.5 μm. This is attributed mainly to the lubricity and cooling provided by these fluids. Additionally, the roughness generated by using the different JO + PDFC emulsions was in the range of that produced by using CIMSTAR^®^. However, some JO + PDFC emulsions presented lower roughness than CIMSTAR^®^, i.e., the emulsion 20%JO + 1%PDFC. Additionally, it was found that the surface roughness increases as the JO concentration rises.

#### 3.2.4. Chip Formation Analysis

**Figure 10:**
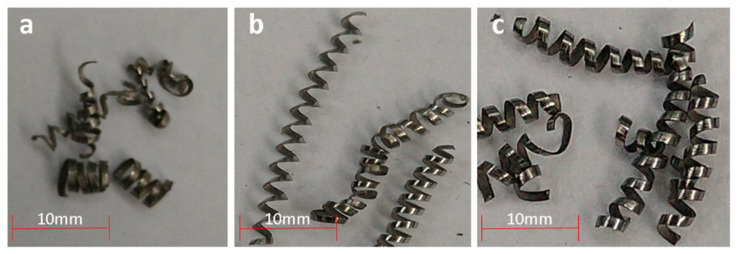
Chip type and quality: (**a**) dry machining; (**b**) CIMSTAR^®^ 60; (**c**) 20% JO + 1% PDFC emulsion.

The type and shape of the chips formed during cutting processes are used as a visual indicator of the effectiveness of the cutting parameters and MWF performance [36,37]. Thus, samples of chips formed during each experiment were analyzed visually. Figure 10a–c show examples of the characteristic chips formed during dry machining, turning using CIMSTAR^®^ and turning using 20%JO + 1%PDFC, respectively. It is noteworthy that all the JO + PDFC emulsions generated similar chips. Dry machining generated a combination of multiple chip types that includes very short (between 7 and 12 mm) snarled conical chips, short tubular chips and ribbon chips. The short tubular chips were formed during the first part of the experiment, while ribbon and snarled chips are an indicator of a non-constant cutting profile, resulting in a poor surface finish, high tool wear and low workpiece accuracy [37]. The turning operations carried out by using CIMSTAR^®^ produced only long (20–35 mm) tubular chips. Although this can be a positive performance indicator (constant cutting profile, low tool wear and good surface finish), this type of long continuous chip is not always recommended since they can easily tangle around the workpiece or the tool can cause scratching of the workpiece surface [38]. In the case of the operations run by suing all the JO + PDFC emulsions, they generated a mix of medium–short (15–30 mm) tubular chips, which are associated with low tool wear and good surface finishing. Technically, they are ideal for high productivity machining because they are very easy to handle and present a low risk of a tangle.

Overall, the emulsions produced with the JO base and PDFC emulsifier exhibited acceptable performance in terms of cutting forces and cutting temperature and also exceptional workpiece’s surface finishing and chip formation compared to dry machining and the use of a commercial WMF. The variability of biopolymer production batches is a constraint on industrial applications; nevertheless, efforts are made on chitin characterization based on their degree of acetylation, molecular weight and chemical composition for standardization on obtaining similar properties regardless of their origin and production process [7]. Similarly, the chemical characteristics of Jatropha oil, in terms of fatty acids, can change according to each crop location and weather; nevertheless, JO composition variations in their tribological behavior do not change considerably. Thus, in general, the origin of these components has minimal influence on the final product properties and performance as a lubricant if they meet the acetylation degree in the case of chitin and have a similar fatty acids’ profile in the case of Jatropha oil. Nonetheless, other vegetable oils (preferable non-edible) can be used as the base oil for the emulsions since the tribological behavior is also convenient [18,21,22,23,24,25]. According to this pioneering exploration on the use of PDFC as an emulsifier for producing green MWFs, it was observed that the JO + PDFC emulsion stability and cooling properties should be enhanced to meet similar, or even, better performance than commercial MWFs. Considering that commercial MWFs are formulated with different additives, which are harmful to the environment, to exhibit superior stabilization and performance, the developed JO + PDFC emulsions can be a potential green option because they are based on only natural biodegradable products without any additive or petroleum derivates. In this sense, to maintain the green attributes and improve the stability and cooling properties of these new classes of MWFs, further emulsion formulations with green additives and emulsion waste treatment are a topic of ongoing research of our group.

## 4. Conclusions

Pickering emulsions of a non-edible JO base and PDFC emulsifier (JO + PDFC) were successfully prepared to be tested as green MWFs. PDFC was produced by biological extraction of chitin from shrimp waste, heads and shells of the species *Litopenaeus vannameii*, followed by partial deacetylation and further fibrillation. The amount of JO had a significant effect on the emulsion stability, owing to its fatty acid composition that interacts with the amine groups of the PDFC. All the JO + PDFC emulsions exhibited significantly lower cutting forces than dry machining and almost similar values to a commercial semisynthetic MWF. Additionally, the cutting forces tended to reduce with the increase in JO concentration in the emulsions, while the variation in PDFC content did not have a significant effect. Comparing all the JO + PDFC emulsions, the best performance as cutting fluid was obtained using 20 wt% of JO with any concentration of PDFC. The use of all the JO + PDFC emulsions did not reduce the cutting interface temperature as effectively as the commercial semisynthetic MWF, and they promoted a minimal temperature change (from +2 up to +3.5 °C), which is positive for turning processes, while the commercial MWF generated a temperature change of about −2 °C. The temperature change increased with the increase in JO contained in the emulsions, while the concentration of PDFC had no significant effect on the temperature change. The workpiece finishing achieved using all the JO + PDFC emulsions was similar to that produced using the commercial semisynthetic MWF. However, the emulsion 20% JO + 1% PDFC generated a better finishing than the commercial MWF. In addition, the operations run by using all the JO + PDFC emulsions generated a mix of medium–short (15–30 mm) tubular chips, which are associated with low tool wear and good surface finishing. Finally, the use of the biodegradable base oil and the biodegradable emulsifier obtained from biomass waste (shrimp waste) is a pioneer attempt at proposing a potential alternative to replace toxic and fossil-based MWFs, which can have a significant impact on achieving cleaner production demands and the utilization of waste biomass contributing with the environmental care.

## Data Availability

Data is contained within the article.
